# Serum Uric Acid to Creatinine Ratio and Risk of Metabolic Syndrome in Saudi Type 2 Diabetic Patients

**DOI:** 10.1038/s41598-017-12085-0

**Published:** 2017-09-21

**Authors:** Nasser M. Al-Daghri, Omar S. Al-Attas, Kaiser Wani, Shaun Sabico, Majed S. Alokail

**Affiliations:** 1 Biomarkers Research Program, Biochemistry Department, College of Science, King Saud University, Riyadh, 11451 Saudi Arabia; 2 Prince Mutaib Chair for Biomarkers of Osteoporosis, Biochemistry Department, College of Science, King Saud University, Riyadh, 11451 Saudi Arabia

## Abstract

This study aimed to investigate whether uric acid to creatinine (UA/Cr) ratio is associated with higher risk of metabolic syndrome (MetS) and its components. 332 adult Saudi type 2 diabetes mellitus (T2DM) patients were divided into UA/Cr tertiles. Risk for full MetS was significantly highest in individuals that constitutes the uppermost serum UA/Cr tertile [Odds ratio (OR): 1.80, 95% confidence interval (CI): 1.0–3.3; p < 0.001) after adjustment for age, gender and BMI. Similarly, risk for individual components of MetS like central obesity, hypertriglyceridemia, low HDL-cholesterol and hypertension was significantly highest in this tertile with OR’s of 2.61 (1.2–5.6), 1.42 (0.7–2.3), 1.45 (0.7–2.8) and 1.16 (0.6–2.2) respectively (all p-values < 0.001) after adjustment for age, gender, BMI and other components of MetS. Furthermore, serum UA/Cr levels increased with increasing number of MetS components (mean values of 4.44, 4.49, 4.64, 4.89 and 4.91 respectively for 1,2,3,4 and 5 MetS components, p-values < 0.001 after adjusting for age, gender and BMI). Our data suggest that serum UA/Cr in T2DM patients is strongly associated with full MetS as well as its individual components. These findings are of considerable clinical importance as serum UA/Cr may be used as a marker in the pathogenesis of MetS.

## Introduction

Metabolic syndrome (MetS) is a major worldwide public health problem and is defined as a cluster of cardiovascular risk factors like obesity, hyperglycemia, hypertriglyceridemia, low high density lipoprotein cholesterol levels and hypertension^1^. In the Arabian Gulf region, the prevalence of MetS varies from 20.7–37.2% in males and from 32.1–42.7% in females^2^. In Saudi Arabia, the age-adjusted prevalence of MetS in males and females was reported to be 37.2% and 42% respectively^3^. The recent rapid economic growth and Westernization of lifestyle were partially blamed for the rise in MetS^4^. MetS is clinically important as it helps identify patients at high risk of developing cardiovascular disease (CVD), type 2 diabetes mellitus (T2DM)^5,6^ and chronic kidney disease (CKD)0^7^.

There is a growing interest in the relationship of MetS with CKD. In a meta-analysis of 11 studies, MetS was reported to be associated with stage III CKD progression^8^. Both MetS and CKD are related to increased CVD events and the effect is cumulative if both are present^9^. However, the earliest stages of CKD are typically difficult to diagnose due to its asymptomatic nature. Biomarkers that could help identify the progression of different components of MetS could provide more effective prevention strategies and may unfold the relationship between these components and pathogenesis of CKD and incident CVD.

Serum creatinine (Cr) is a commonly used indicator for detecting small changes in glomerular filtration rate (GFR), hence a good biomarker of early stage CKD^10^. Raised circulating levels of Cr was found to be associated with increased risk of CVD, obesity and hypertension^11–13^. Similarly, elevated levels of serum uric acid (UA) is reported to be a marker for decreased renal function^14^ and a risk factor for hypertension and CVD^15,16^. Studies also suggest that serum UA is a strong indicator for type 2 diabetes mellitus (T2DM) independent of other confounding factors^17^. There is however limited evidence that these serum biomarkers could be used to detect pathogenesis of MetS. In this study, we combined these two biomarkers in ‘serum uric acid to creatinine ratio’ (UA/Cr). This biomarker is studied before^18–20^, however it is yet to be studied in relation to metabolic syndrome and in a population where the prevalence of MetS and T2DM are high^21,22^. This study aimed to fill that gap and investigate association of serum UA/Cr with MetS and its components in Saudi T2DM patients.

## Results

### General characteristics of subjects according to serum UA/Cr tertiles

A total of 332 Saudi subjects with T2DM were recruited for in this cross-sectional study. The mean age of the study population was 52.94 ± 11.4; 40.4% of whom were males. The overall prevalence of MetS was 56.3%. Subjects were divided into serum UA/Cr tertiles (Ter). Ter1 ranged from 2.1 to 4.0 [N = 110, mean ± standard deviation (SD) 3.24 ± 0.5], Ter2 ranged from 4.1 to 5.0 (110, 4.50 ± 0.3) and Ter3 ranged from 5.1 to 11.1 (112, 6.16 ± 1.0). The results are presented in Table 1. Individuals in the highest tertile of serum UA/Cr were significantly younger (50.1 years in Ter3, 53.92 in Ter2 and 54.84 in Ter1, p = 0.005) and had higher BMI (33.9 in Ter3, 32.17 in Ter2 and 30.41 in Ter1, p < 0.001)) than the ones in lowest tertile. The prevalence of MetS significantly increased in parallel with increasing tertiles, from 42.9% (Ter1) and 59.8% (Ter2) to 67.6% (Ter3) (p = 0.003). The differences in the prevalence of individual components of MetS from highest versus lowest tertiles of serum UA/Cr was statistically significant in central obesity (64.4% in Ter3 vs. 25.5% in Ter1, p < 0.001) and low HDL-cholesterol (64.8% in Ter3 vs. 48.6% in Ter1, p = 0.001). With respect to metabolic parameters, individuals in the higher serum UA/Cr tertile had significantly higher BMI, waist and hip circumferences, insulin, HOMA-IR and eGFR (all p < 0.05). In contrast, the individuals with higher serum UA/Cr tertile showed lower levels of fasting glucose, total cholesterol, HDL-cholesterol and urea (all p-values < 0.05).

**Table 1:** General Characteristic of Subjects based on Serum UA/Cr Tertiles.

	Ter 1 (2.1–4.0)	Ter 2 (4.1–5.0)	Ter 3 (5.1–11.1)	P-Value	Total
N	110	110	112		332
UA/Cr	3.2 ± 0.5	4.5 ± 0.3	6.2 ± 1.0	<0.001	4.6 ± 1.3
**Clinical Characteristics**					
Age (years)	54.8 ± 11.4	53.9 ± 10.3	50.1 ± 12	0.005	52.9 ± 11.4
BMI (kg/m²)	26.8 ± 4.7	28.31 ± 4.3	29.8 ± 5.1	<0.001	28.4 ± 4.9
Waist (cm)	94.5 ± 10.2	95.7 ± 9.1	96.55 ± 10.2	0.04	95.7 ± 9.8
Hips (cm)	97.2 ± 12.2	99.4 ± 11.9	104.4 ± 10.9	0.001	100.4 ± 11.9
SBP (mmHg)	135.5 ± 16.9	138.1 ± 16.5	139.0 ± 13.5	0.92	137.6 ± 15.7
DBP (mmHg)	79.8 ± 9.1	81.6 ± 8.9	82.12 ± 8.2	0.92	81.2 ± 8.8
**Glycemic Profile**					
Fasting Glucose (mmol/l)^#^	9.43 (7.8, 12.1)	8.90 (7.2, 11.4)	8.1 (6.6, 10.0)	0.02	9.01 (7.1, 11.3)
Insulin (µlU/ml)^#^	11.8 (6.6, 17.1)	13.3 (6.7, 20.5)	14.1 (8.9, 21)	0.04	13.14 (7.2, 20.8)
HOMA-IR^#^	4.5 (2.9, 7.6)	4. 9 (2.7, 8.5)	5.2 (2.9, 8.1)	0.04	4.83 (2.9, 8.2)
**Lipid Profile**					
Total Chol (mmol/l)	5.2 ± 1.1	4.8 ± 1	5.1 ± 1.1	0.03	5.05 ± 1.1
Triglycerides (mmol/l)	1.7 ± 0.9	1.7 ± 0.9	1.6 ± 0.7	0.81	1.66 ± 0.8
HDL-Chol (mmol/l)	1.2 ± 0.4	1.1 ± 0.3	1.1 ± 0.3	0.006	1.14 ± 0.3
**Renal Profile**					
Calcium (mmol/l)	2.5 ± 0.3	2.5 ± 0.3	2.5 ± 0.2	0.82	2.5 ± 0.3
Albumin (g/l)	42.0 ± 4.4	42.4 ± 5.2	41.0 ± 4.7	0.07	41.8 ± 4.8
Uric Acid (µmol/l)	239.5 ± 61.6	273.2 ± 62.5	338.3 ± 94.5	0.001	283.8 ± 84.9
Creatinine (µmol/l)	76.3 ± 22.6	61.4 ± 13.9	55.6 ± 12.6	0.001	64.39 ± 19
eGFR (ml/min/1.73²)	93.9 ± 24.3	110.8 ± 23.8	122.5 ± 30.3	0.001	109.1 ± 28.8
UREA (mmol/l)	5.2 ± 1.5	4.6 ± 1.7	4.6 ± 1.3	0.003	4.81 ± 1.5
**MetS Components (%)**					
Central Obesity	25.5	54.3	64.4	<0.001	48.1
Hyperglycemia	100	100	100	1.0	100
High TG	29.9	32.7	34.3	0.853	32.9
Low HDL-C	48.6	62.6	64.8	0.001	58.1
Hypertension^$^	49.5	48.6	54.7	0.749	51.4
MetS	42.9	59.8	67.6	0.003	56.3

Note: Data presented as mean ± standard deviation for normal variables and median (Q1, Q3) for non-normal variables (#). P-values derived from one way ANOVA (Kruskal-Wallis test for non-normal). SBP is systolic blood pressure, DBP is diastolic blood pressure, Chol is cholesterol and eGFR is estimated glomerular filtration rate.

### Association between serum UA/Cr and other measured parameters

Correlations between serum UA/Cr and other measured parameters are summarized in Table 2. The first column shows unadjusted correlation coefficients and the corresponding p-value while r^a^ in the second column shows correlation coefficients after adjustment with age, gender and BMI. Serum UA/Cr is inversely correlated with age (r = −0.175, p = 0.002), fasting glucose (r = −0.131, p = 0.02) and HDL-cholesterol (r = −0.175, p = 0.002).A significant positive correlation was found between serum UA/Cr and BMI (r = 0.214, p < 0.001), waist circumference (r = 0.135, p = 0.04), hip circumference (r = 0.234, p < 0.001) and eGFR (r = 0.359, p < 0.001).

**Table 2:** Correlation between Serum UA/Cr and other measured parameters in T2DM.

Parameters	r	p	r^a^	p^a^
Age (years)	−0.205	<0.001	−0.175	0.002
BMI (kg/m^2^)	0.324	<0.001	0.214	<0.001
Waist (cm)	0.146	0.05	0.135	0.04
Hips (cm)	0.319	<0.001	0.234	<0.001
SBP (mmHg)	−0.017	0.76	−0.030	0.60
DBP (mmHg)	−0.001	0.99	−0.017	0.77
Fasting Glucose (mmol/l)^#^	−0.138	0.01	−0.131	0.02
Insulin (µlU/ml)^#^	0.083	0.14	0.075	0.18
HOMA-IR^#^	−0.017	0.76	−0.024	0.67
Total Cholesterol (mmol/l)	−0.067	0.23	−0.091	0.10
Triglycerides (mmol/l)	−0.030	0.59	0.003	0.95
HDL-Cholesterol (mmol/l)	−0.133	0.02	−0.175	0.002
Calcium (mmol/l)	0.005	0.92	0.060	0.29
Albumin (g/l)	−0.121	0.03	−0.044	0.44
eGFR (ml/min/1.73²)	0.412	<0.001	0.359	<0.001
Urea (mmol/l)	−0.114	0.04	−0.013	0.81

Note: r and p are the unadjusted correlation coefficients and associated p-value. r^a^ and p^a^ are values after adjustment with age, gender and BMI. SBP is systolic blood pressure, DBP is diastolic blood pressure and eGFR is estimated glomerular filtration rate. Non-normal variables (#) are log-transformed before analysis.

### Association of serum UA/Cr with metabolic syndrome and its components

**Figure 1:**
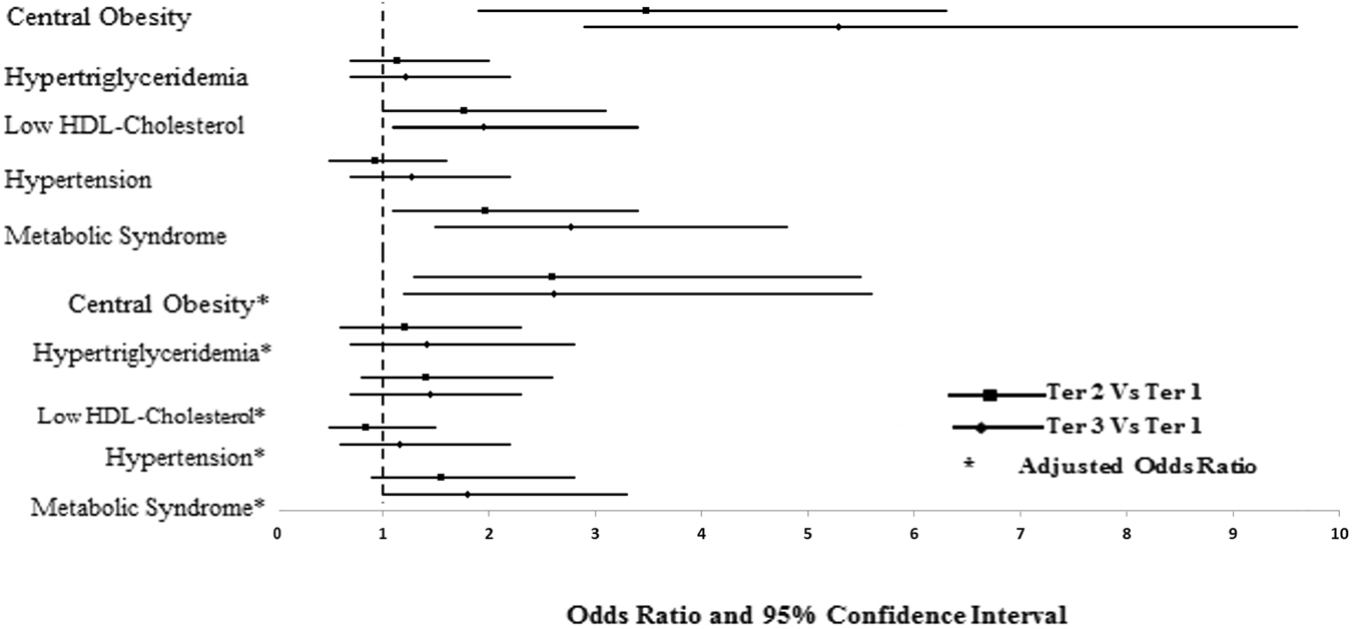
Odds ratio (OR) of MetS and its components in individuals with higher SrUa/Cr tertiles compared to lowest one. Central obesity [waist circumference >101.6 cm (males), >88.9 cm (females)], hypertriglyceridemia (triglycerides ≥1.7 mmol/l), low high-density lipoprotein cholesterol [HDL-cholesterol <1.03 (males), <1.30 (females)], hypertension (systolic blood pressure >130 mmHg and/or diastolic blood pressure >85 mmHg or current use of antihypertensive medications). Lowest serum UA/Cr tertile is taken as reference to calculate Odds Ratio. For each MetS component, values are adjusted for age, gender, BMI and other MetS components taken as dichotomized variable. The dotted line shows reference (lowest UA/Cr tertile).

Table 3 presents the associations of serum UA/Cr levels and MetS risk and its components. Individuals with the highest serum UA/Cr tertile had higher odds of having full MetS compared with those in lowest tertile (Ter3 vs Ter1-OR: 1.80, 95% CI: 1.0 to 3.3; Ter2 vs Ter1-OR: 1.55, 95% C.I. 0.9 to 2.8; p for trend <0.001) after adjustment for age, gender and BMI. Associations of serum UA/Cr with individual components of MetS also revealed a similar trend. The odds ratio (OR.) of central obesity was 2.61 (C.I. 1.2–5.6) and 2.60 (1.3–5.5) with p < 0.001 for the trend in ter3 and ter2 respectively when compared to the lowest tertile (ter1) after adjustment with age, gender, BMI and rest of the components of MetS taken as dichotomized variables. Similarly, the odds ratios (OR) after adjustments for hypertriglyceridemia, low HDL-cholesterol and hypertension in ter3 vs. ter1 was 1.42 (0.7–2.8), 1.45 (0.7–2.3) and 1.16 (0.6–2.2) respectively; and ter2 vs. ter1 was 1.21 (0.6–2.3), 1.41 (0.8–2.6) and 0.84 (0.5–1.5), respectively (p-values < 0.001). Hyperglycemia, as a component of MetS, was excluded from the analysis since all subjects had fasting glucose >5.6 mmol/l. The odds ratio of MetS and its components in the higher tertiles (2 and 3) of serum UA/Cr compared to the lowest tertile (1) is shown in Fig. 1.

**Table 3:** Adjusted Odds ratios for MetS and components in Serum UA/Cr Tertiles.

Serum UA/Cr	OR (95% CI)			P-value
	Tertile 1	Tertile 2	Tertile 3	
N	107	107	108	
**Central Obesity**				
Model a	Reference	3.48 (1.9–6.3)	5.29 (2.9–9.6)	<0.001
Model b	Reference	3.46 (1.9–6.2)	5.12 (2.8–9.4)	<0.001
Model c	Reference	2.88 (1.4–5.7)	3.28 (1.6–6.6)	<0.001
Model d	Reference	2.55 (1.2–5.3)	2.54 (1.2–5.4)	<0.001
Model e	Reference	2.60 (1.3–5.5)	2.61 (1.2–5.6)	<0.001
**Hypertriglyceridemia**				
Model a	Reference	1.14 (0.7–2.0)	1.22 (0.7–2.2)	0.787
Model b	Reference	1.14 (0.7–2.0)	1.19 (0.7–2.1)	0.026
Model c	Reference	1.19 (0.7–2.1)	1.29 (0.7–2.4)	<0.001
Model d	Reference	1.21 (0.7–2.2)	1.38 (0.8–2.6)	<0.001
Model e	Reference	1.21 (0.6–2.3)	1.42 (0.7–2.8)	<0.001
**Low HDL-Cholesterol**				
Model a	Reference	1.77 (1.0–3.1)	1.95 (1.1–3.4)	0.033
Model b	Reference	1.76 (1.0–3.0)	1.80 (1.0–3.1)	0.003
Model c	Reference	1.63 (0.9–2.8)	1.68 (0.9–2.9)	<0.001
Model d	Reference	1.58 (0.9–2.8)	1.62 (0.9–2.7)	<0.001
Model e	Reference	1.41 (0.8–2.6)	1.45 (0.7–2.3)	<0.001
**Hypertension**				
Model a	Reference	0.93 (0.5–1.6)	1.27 (0.7–2.2)	0.480
Model b	Reference	0.94 (0.6–1.6)	1.42 (0.8–2.5)	0.010
Model c	Reference	0.87 (0.5–1.5)	1.23 (0.7–2.2)	<0.001
Model d	Reference	0.79 (0.4–1.4)	1.05 (0.6–1.9)	<0.001
Model e	Reference	0.84 (0.5–1.5)	1.16 (0.6–2.2)	<0.001
**MetS**				
Model a	Reference	1.97 (1.1–3.4)	2.77 (1.5–4.8)	0.001
Model b	Reference	1.96 (1.1–3.4)	2.67 (1.5–4.7)	<0.001
Model c	Reference	1.65 (0.9–2.9)	2.11 (1.1–3.6)	<0.001
Model d	Reference	1.55 (0.9–2.8)	1.80 (1.0–3.3)	<0.001

Note: Significance was set at p < 0.05. Model ‘a’ is unadjusted (univariate). Each Model is adjusted for same set of variables in the previous model plus age (continuous), gender (male, female), BMI (continuous) and other components of MetS as present/absent in models ‘b’, ‘c’, ‘d’, and ‘e’ respectively.

The odds ratio of MetS and its components vis-à-vis different tertiles of serum UA/Cr after stratification for sex showed a similar trend observed in the overall data (Supplementary file 1).

**Figure 2:**
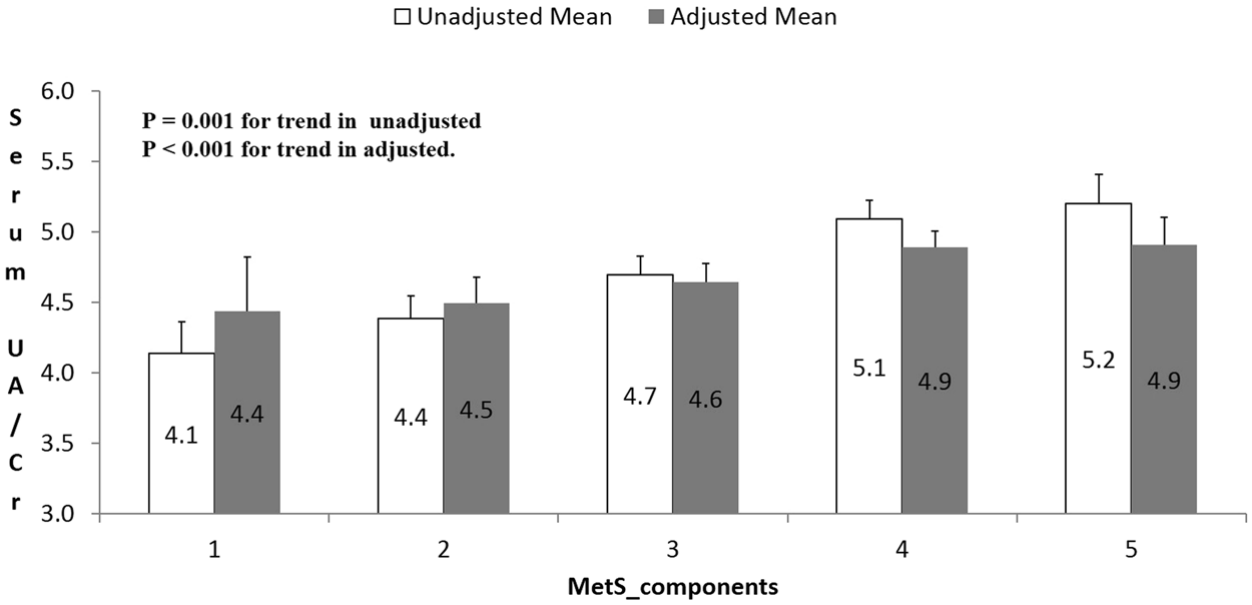
Serum UA/Cr levels in individuals according to number of MetS components. Values are adjusted for age, gender and BMI. The adjusted mean (standard error of mean) serum UA/Cr value for the subjects with 1, 2, 3, 4 and 5 MetS components is 4.44 (0.2), 4.49 (0.1), 4.64 (0.1), 4.89 (0.2) and 4.91 (0.4) respectively.

Interestingly, serum UA/Cr levels increased in parallel with the number of MetS components (Fig. 2). The mean (standard error of mean) serum UA/Cr value for the subjects with 1,2,3,4 and 5 MetS components was 4.44 (0.2), 4.49 (0.1), 4.64 (0.1), 4.89 (0.2) and 4.91 (0.4) respectively with p-value < 0.001 for the trend after adjustment with age, gender and BMI.

## Discussion

To the best of our knowledge, this is the first study to demonstrate the association of serum uric acid to creatinine ratio with MetS and its components. Earlier studies^18,19^ showed that higher serum UA/Cr correlated with lower levels of FEV1/FVC (Tiffeneau-Pinelli index) which is used in the diagnosis of chronic obstructive pulmonary disease (COPD). Another study by Liubao and colleagues^20^ suggested serum UA/Cr might be a better predictor of incident CKD than serum UA alone.

Higher serum UA/Cr levels correlated with an increased risk in all MetS components. The odds of having central obesity, hypertriglyceridemia, low HDL-cholesterol and hypertension for the highest serum UA/Cr tertile compared to the lowest were significant at 2.61, 1.42, 1.45 and 1.16 respectively (p < 0.001 in all) even after adjustment for age, gender, BMI and the rest of MetS components. Hyperuricemia was shown to be significantly associated with higher BMI, dyslipidemia and hypertension in earlier reports^23,24^. The underlying mechanism is still not well understood, though insulin resistance is suspected to be the mechanism interlinking hyperuricemia with the development of these metabolic disorders. Recently, the components of MetS were also found to be associated with high serum Cr levels^25,26^. In our study, higher BMI, waist and hip circumference was observed in the highest tertile of serum UA/Cr which was confirmed by a strong positive correlation of serum UA/Cr with BMI, waist and hips. In contrast, HDL-cholesterol was significantly lowest in the highest tertile of serum UA/Cr and confirmed by the significant inverse correlation of SrUa/Cr with HDL-cholesterol. Notably, levels of serum UA/Cr increased in subjects having more cradiometabolic risk factors and hence it may be useful in determining prognosis for MetS.

An interesting finding in this study is the inverse relation of fasting glucose with serum UA/Cr. Fasting glucose decreased from lower to higher serum UA/Cr tertiles and was also found to be inversely correlated with serum UA/Cr. Chronic high fasting glucose in T2DM subjects promotes hyper filtration state resulting in increased renal excretion of uric acid^27^ and this partially explains the inverse relationship, though we don’t have the data on the duration of diabetes in these subjects.

The association between serum UA and full MetS has been studied in other populations^28,29^. Similarly, a recent study^30^ showed serum Cr to be associated with MetS. In this study, we observed that having MetS was substantially higher in the higher tertiles of serum UA/Cr than the lower ones (Table 3). The prevalence of MetS also increased from lower to higher serum UA/Cr tertiles (Table 1). This can be partially explained by hyperuricemia derived endocrine imbalance in adipose tissue eventually resulting in low grade inflammation found in MetS^31^. It has also been observed that UA stimulates production of pro-inflammatory cytokines like C-reactive protein and tumor necrosis factor α^32^ and hence could potentially modulate chronic inflammatory processes. A noticeable increase in intake of fructose and purine-rich diet in the past few decades may contribute to raised serum uric acid levels and correlates well with rising prevalence of MetS^33^.

Interestingly, renal function is shown to be the main confounding factor for the association of serum UA with MetS and its components^24,28,29^. UA, which is a final product of purine metabolism, is mainly eliminated in the urine. Hence, impaired renal function associated with lower eGFR and higher serum creatinine levels correspond to higher levels of serum UA. Serum UA, in this study, is significantly and positively correlated with creatinine. Elevated serum UA may indicate an altered kidney function which itself has been found to be an independent predictor of MetS and CVD events^34,35^. Renal function-normalized serum UA such as serum UA/Cr, reflecting the net production of UA, may thus turn out to be a good marker in pathogenesis of MetS and related diseases.

The authors acknowledge some limitations. First, due to the cross-sectional nature of our study it is difficult to infer causality between risk of MetS and levels of serum UA/Cr. Secondly, although the data was adjusted for some confounding variables like age, gender, BMI and presence/absence of individual MetS components, we don’t have sufficient information on some other factors like duration of diabetes, menopausal status or dietary habits to include in our analysis. Residual confounding by these unknown factors is a possibility, although it seems unlikely that the strength of observed association between serum UA/Cr and MetS is completely nullified by these factors. Nevertheless, the study is the first to provide insights as to the role of serum UA/Cr in assessing MetS risk in a homogenous Arab population with T2DM. Future prospective studies taking into account the mentioned confounding factors should be designed to explore the causality and expand on the present findings.

## Conclusion

In conclusion, our data suggest that levels of serum UA/Cr in T2DM are strongly associated with risk of MetS and its components even after adjusting for age, gender, and BMI. Consequently, lowering serum UA/Cr levels by adopting a healthier lifestyle may prove to be a useful strategy for lowering MetS burden. Further research is needed to address the causal relationship of serum UA/Cr in the pathogenesis of MetS.

## Methods

### Subjects, Anthropometry and Blood Collection

A total of 332 adult Saudi subjects with T2DM were selected randomly from Riyadh 2 cohort, whose details are previously published^22^. In brief, the subjects were recruited as a part of a nationwide screening project conducted by Biomarkers Research Program (BRP) at King Saud University, Riyadh, Saudi Arabia. A standardized manual of operation was followed for collecting fasting blood samples and the questionnaire data from each participant at the respective health center. Exclusion criteria include pregnant women and those with known complications such as renal, hepatic and cardiovascular diseases. Also, those taking medications related to these diseases and the ones that affect the circulating levels of uric acid like aspirin, thiazide diuretics etc. were excluded. Anthropometry included height, waist and hips (cm) and weight (kg) measured using Digital Pearson Scale (ADAM equipment Inc., USA). A standard procedure was utilized to measure resting blood pressure (measured twice by a qualified nurse). Blood samples were collected into a non-heparinized tube for centrifugation. The resulting serum samples were transferred immediately to the analysis facility; divided into several aliquots and stored at −80 °C until analysis.

Prior to inclusion, all the participants provided written and informed consent. The study was approved by the Ethics Committee of the College of Science, King Saud University, Riyadh, Saudi Arabia.

### Biochemical measurements

All biochemical parameters except insulin were measured using a standard biochemical analyzer (Konelab, Thermo-Fisher Scientific, Finland). Serum insulin was quantified using fluorescent microbead technology by Luminex multiplex (Luminexcorp, TX, USA). Fasting glucose and lipid profile was quantified using routine biochemical tests in Konelab. The limit of detection for the assay was 0.02 mmol/l, 0.1 mmol/l, 0.04 mmol/l and 0.02 mmol/l for glucose, total cholesterol, HDL-cholesterol and triglyceride assays respectively. Calcium assay utilized Arsenazo III and albumin assay utilized a specific dye called bromocresol purple.

Cr, UA and urea assays were quantified using an enzymatic method utilizing creatinase, uricase and urease respectively supplied in ready to use reagents from Thermo-Fischer (reference numbers 981845,981788,981820 respectively). The limit of detection was 2μmol/l, 3.4 μmol/l and 1.1 mmol/l respectively. The imprecision of the creatinine, uric acid and urea assays was less than 1.4%, 4% and 6% respectively of the total CV. The calibrators and controls supplied were used routinely to check the performance of the assays. The QA department of King Saud University audits the BRP laboratory at regular intervals.

HOMA-IR was calculated as serum insulin (µlU/ml) x fasting glucose (mmol/l) /22.5^36^. To estimate glomerular filtration rate (eGFR), we used modification of diet in renal disease (MDRD) equation which is:

$$\begin{array}{lcc}eGFR\,(in\,ml/min/1.73\,{m}^{2}) & \,= & \,{186}\,\times \,{[SCR/88.4]}^{-{1.154}}\times \,{[ageinyears]}^{-{0.203}}\\  &  & \,\times \,[{0.742}\,if\,female]\,\times \,{1.210}\,(if\,black)\end{array}$$

*where SCR is serum creatinine in μmol/l* ^37^.

### Definition of MetS

The National Cholesterol Education Programme Adult Treatment Panel III (NCEP ATP III) criteria was used to define MetS present if having three out of five components^38^. The five components of MetS tested in this study were as follows

1. Central Obesity: Waist circumference >101.6 cm (males), >88.9 cm (females).
2. Hyperglycemia: Fasting glucose >5.6 mmol/l or pharmacologic treatment for hyperglycemia.
3. Hypertriglyceridemia: Serum triglycerides ≥1.7 mmol/l.
4. Low HDL-Cholesterol: Serum HDL-cholesterol <1.03 mmol/l (males), <1.30 mmol/l (females).
5. Hypertension: Systolic blood pressure >130 mmHg and/or diastolic blood pressure >85 mmHg or current use of antihypertensive medications.

### Data analysis

SPSS version 16.0 (Chicago, IL, USA) was used to analyze data. For statistical analysis, subjects were divided into serum UA/Cr tertiles. Tertiles 1, 2 and 3 ranged from 2.1 to 4.0 (N = 110), 4.1 to 5.0(N = 110) and 5.1 to 11.1(N = 112), respectively. Normal continuous variables were presented as mean ± standard deviation and non-normal variables were presented as median (first quartile, third quartile). Nominal variables were presented as percentages (%). Appropriate statistical tests were employed to check differences in central tendency as a trend between serum UA/Cr tertiles. Non-normal variables were log transformed before further analysis. Pearson correlation was used to determine associations between serum UA/Cr and other measured parameters controlling for age, gender and BMI. Multi-nominal logistic regression was done using serum UA/Cr tertiles as dependent variables (lowest tertile as reference) and MetS components (present versus absent) as independent factors with age, gender and BMI as covariates to calculate odds ratio. Significance was set at p < 0.05. Microsoft Excel 2010 was used to prepare figures.

### Data Availability

The dataset analysed during the current study is available from the corresponding author on reasonable request.

### Statement of Human and Animal Rights

All procedures followed were in accordance with the ethical standards of the responsible committee on human experimentation (institutional and national) and with the Helsinki Declaration of 1975, as revised in 2008. The study was approved by the Ethics Committee of College of Science, King Saud University.

## Electronic supplementary material

Supplementary Information
