# Supplementary material for: Serum Uric Acid to Creatinine Ratio and Risk of Metabolic Syndrome in Saudi Type 2 Diabetic Patients
Source: Sci Rep. 2017 Sep 21;7:12104. doi: 10.1038/s41598-017-12085-0 (PMC5608718; doi:10.1038/s41598-017-12085-0)

Serum Uric Acid to Creatinine Ratio and Risk of Metabolic Syndrome in Saudi Type 2 Diabetic Patients

Nasser M Al-Daghri, Omar S. Al-Attas, Kaiser Wani, Shaun Sabico, Majed S. Alokail

Supplementary table 1(a): Adjusted Odds ratio (95% confidence interval) for metabolic syndrome and its components in Serum UA/Cr tertiles in males.

		OR (95% CI)		p value (trend)
Serum UA/Cr	Tertile 1<br>2.1 to 3.4	Tertile 2<br>3.5 to 4.5	Tertile 3<br>4.6 to 9.7	
N	45	45	45	
Central Obesity				
Model a	Reference	3.12 (0.8 - 5.4)	5.54 (1.5 - 10.1)	0.028
Model b	Reference	3.09 (0.8 - 5.4)	5.26 (1.4 - 10.2)	0.081
Model c	Reference	2.07 (0.4 - 5.1)	2.37 (0.6 - 9.5)	0.008
Model d	Reference	2.19 (0.4 - 5.3)	2.68 (0.6 - 10.0)	0.007
Hypertriglyceridemia				
Model a	Reference	1.11 (0.4 - 2.7)	1.60 (0.7 - 3.8)	0.547
Model b	Reference	1.11 (0.4 - 2.7)	1.57 (0.6 - 3.8)	0.772
Model c	Reference	1.13 (0.4 - 2.6)	1.48 (0.5 - 3.7)	0.017
Model d	Reference	1.08 (0.4 - 2.9)	1.49 (0.6 - 3.9)	0.027
Low HDL-Cholesterol				
Model a	Reference	1.21 (0.5 - 2.8)	1.83 (0.8 – 4.2)	0.361
Model b	Reference	1.19 (0.5 - 2.8)	1.77 (0.7 – 4.1)	0.651
Model c	Reference	1.05 (0.4 - 2.5)	1.34 (0.5 - 3.3)	0.021
Model d	Reference	1.21 (0.5 - 3.1)	1.32 (0.5 - 3.4)	0.027
Hypertension				
Model a	Reference	0.62 (0.2 - 1.4)	0.79 (0.3 - 1.8)	0.555
Model b	Reference	0.84 (0.4 - 1.9)	1.21 (0.7 – 2.3)	0.024
Model c	Reference	0.86 (0.5 - 1.6)	1.16 (0.7 - 1.9)	0.026
Model d	Reference	0.79 (0.4 - 1.3)	1.11 (0.5 - 1.9)	0.027
MetS				
Model a	Reference	1.33 (0.6 - 3.1)	1.39 (0.6 - 3.2)	0.004
Model b	Reference	1.32 (0.6 - 3.1)	1.37 (0.6 - 3.2)	0.042
Model c	Reference	1.21 (0.5 - 2.8)	1.32 (0.7 - 3.1)	0.026

Supplementary table 1(b): Adjusted Odds ratio (95% confidence interval) for metabolic syndrome and its components in Serum UA/Cr tertiles in females.

		OR (95% CI)		p value (trend)
Serum UA/Cr	Tertile 1 2.4 to 4.3	Tertile 2 4.4 to 5.5	Tertile 3 5.6 to 11.1	
N	66	66	65	
Central Obesity				
Model a	Reference	2.09 (1.0 - 4.5)	2.29 (1.1 - 5.0)	<0.001
Model b	Reference	2.15 (0.9 - 4.7)	2.29 (1.0 - 5.0)	<0.001
Model c	Reference	2.08 (0.9 - 4.8)	2.62 (1.1 - 6.1)	<0.001
Model d	Reference	2.08 (0.9 - 4.9)	2.86 (1.2 - 6.8)	<0.001
Hypertriglyceridemia				
Model a	Reference	1.17 (0.5 - 2.5)	1.55 (0.7 - 3.3)	0.506
Model b	Reference	1.13 (0.5 - 2.4)	1.55 (0.7 - 3.2)	0.021
Model c	Reference	1.18 (0.5 - 2.6)	1.56 (0.7 - 3.3)	<0.001
Model d	Reference	1.45 (0.6 - 3.4)	2.32 (1.0 - 5.4)	<0.001
Low HDL-Cholesterol				
Model a	Reference	0.94 (0.5 - 1.9)	1.64 (0.8 – 3.4)	0.103
Model b	Reference	1.01 (0.5 - 1.9)	1.63 (0.7 – 3.4)	0.012
Model c	Reference	1.05 (0.7 - 2.1)	1.57 (0.7 - 3.3)	<0.001
Model d	Reference	1.41 (0.6 - 3.1)	1.95 (0.5 - 2.2)	<0.001
Hypertension				
Model a	Reference	1.56 (0.7 - 3.1)	1.66 (0.8 - 3.3)	0.301
Model b	Reference	1.68 (0.8 - 3.4)	1.67 (0.8 - 3.4)	0.081
Model c	Reference	1.64 (0.8 - 3.4)	1.85 (0.9 - 3.8)	0.029
Model d	Reference	1.92 (0.8 - 4.2)	2.15 (0.9 - 4.5)	0.004
MetS				
Model a	Reference	2.64 (1.1 - 6.1)	3.42 (1.4 - 8.1)	0.009
Model b	Reference	2.57 (1.1 - 5.8)	3.47 (1.5 - 8.3)	0.004
Model c	Reference	2.64 (1.1 - 5.9)	3.89 (1.6 - 9.1)	0.008

Note for supplementary table 1(a) and 1(b): Significance was set at $p < 0.05$. Model ‘a’ is unadjusted (univariate). Each Model is adjusted for same set of variables in the previous model plus age (continuous), BMI (continuous) and other components of MetS as present/absent in models ‘b’, ’c’, and ’d’ respectively.

Supplementary figure 1: Odds ratio (OR) of MetS and its components in individuals with higher SrUa/Cr tertiles compared to lowest one (Separately done for males and females). The dotted line shows reference (lowest UA/Cr tertile).

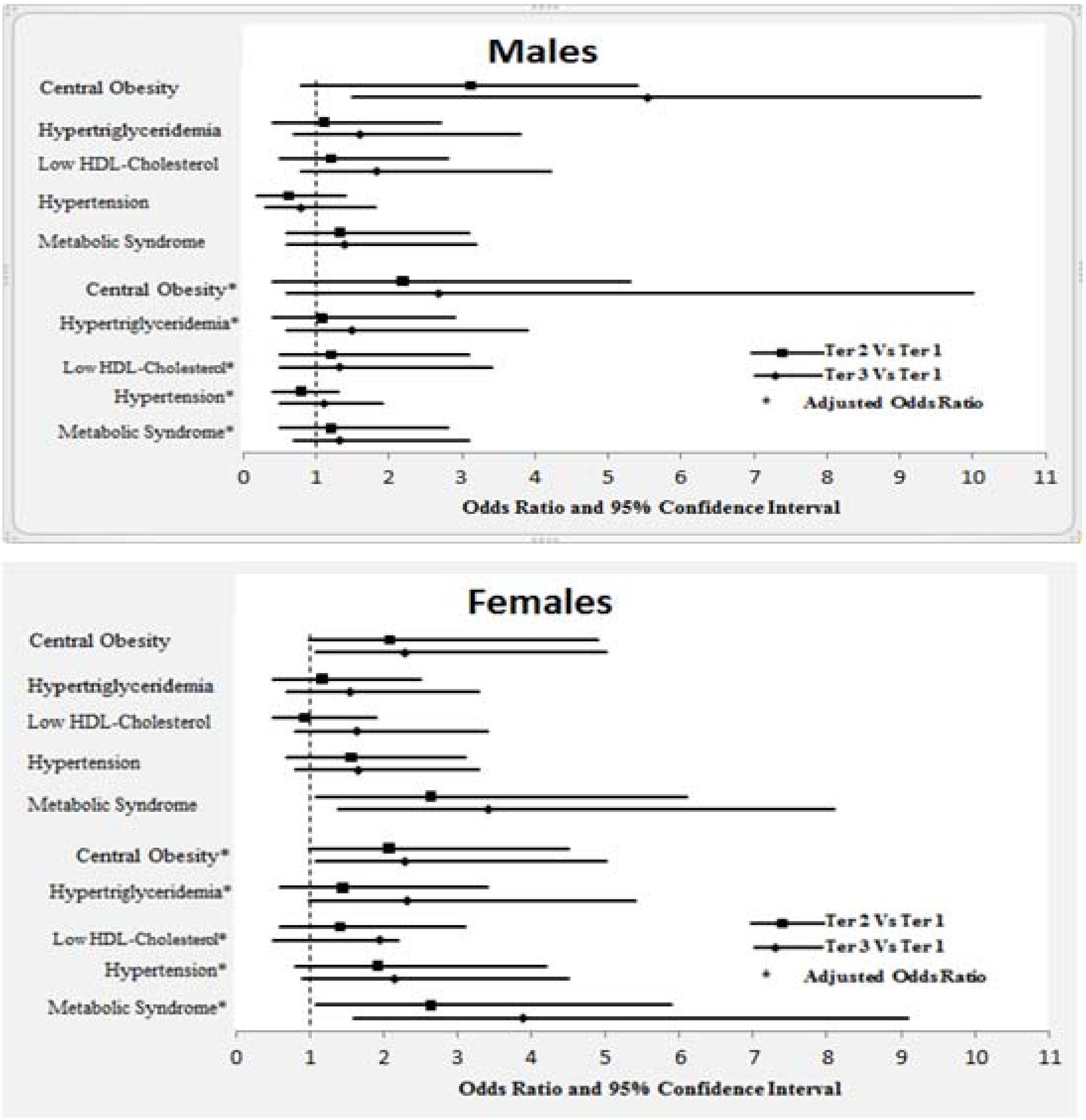

Supplement: Supplementary file 1 — Supplementary Information [file 41598_2017_12085_MOESM1_ESM.pdf]
